# Associations between Th1-related cytokines and complicated pediatric appendicitis

**DOI:** 10.1038/s41598-024-53756-z

**Published:** 2024-02-26

**Authors:** Matilda Elliver, Martin Salö, Bodil Roth, Bodil Ohlsson, Lars Hagander, Johanna Gudjonsdottir

**Affiliations:** 1 Department of Clinical Sciences in Lund, Lund University, Lund, Sweden; 2 Department of Pediatric Surgery, Skåne University Hospital, Lund, Sweden; 3 Department of Clinical Sciences in Malmö, Lund University, Lund, Sweden; 4 Department of Internal Medicine, Skåne University Hospital, Malmö, Sweden; 5 Department of Surgery, Skåne University Hospital, Malmö, Sweden

**Keywords:** Paediatric research, Diagnostic markers, Predictive markers, Acute inflammation, Gastrointestinal system

## Abstract

The pathogenesis of appendicitis is not understood fully, and the diagnosis can be challenging. Previous research has suggested an association between a T helper (Th) 1-dependent immune response and complicated appendicitis. This prospective cohort study aimed to evaluate the association between serum concentrations of the Th1-associated cytokines interleukin (IL)-1α, IL-1β, IL-2, IL-6, IL-10, IL-17A and tumor necrosis factor beta (TNF-β) and the risk of complicated appendicitis in children. Appendicitis severity was determined through histopathological examination. A total of 137 children < 15 years with appendicitis were included with a median age of 10 years (IQR 8–12); 86 (63%) were boys, and 58 (42%) had complicated appendicitis. Children with complicated appendicitis had significantly higher concentrations of serum IL-6 and IL-10, and lower of TNF-β. After adjustment for age, symptom duration, and presence of appendicolith in a multivariable logistic regression, a higher concentration of IL-6 remained associated with an increased risk of complicated appendicitis (aOR 1.001 [95% CI 1.000–1.002], *p* = 0.02). Serum concentrations of IL-1α, IL-1β, IL-2, IL-10, IL-17A and TNF-β were not significantly associated with the risk of complicated appendicitis. In conclusion, our results suggests that the systemic inflammatory response in complicated appendicitis is complex and not solely Th1-dependent.

## Introduction

Appendicitis is the leading cause for children to undergo emergency abdominal surgery^1^. Despite this, the pathogenesis of appendicitis is not understood fully^2^. For a long time, it was believed that appendicitis inevitably progressed to perforation and peritonitis, which is why early surgery for all cases was advocated, and high rates of negative appendectomies were accepted. However, it has become evident that not all cases of appendicitis progress to perforation and that many cases of simple appendicitis heal spontaneously^3–5^. Consequently, it has been suggested that appendicitis should be divided into two entities: uncomplicated and complicated appendicitis. Uncomplicated appendicitis may be self-limiting, but might reoccur, whereas complicated appendicitis progresses rapidly and causes various complications if not treated in good time. Hence, it is of considerable clinical importance to differentiate between these two entities of appendicitis and to identify children who will benefit from surgery.

It has been proposed that a person’s immune system affects the disease course towards uncomplicated or complicated appendicitis. This hypothesis stems from epidemiological studies^6–8^, but also from clinical studies that have suggested that complicated appendicitis is associated with a T helper (Th)1-dependent and Th17-dependent immune response, whereas uncomplicated appendicitis is associated with a Th2-dependent immune response^9,10^. Furthermore, children with IgE-mediated allergy, a Th2-driven condition, carry up to three times a lower risk of complicated appendicitis compared to non-allergic children^11,12^. In a recent study, we hypothesized that higher serum concentrations of Th-2 associated interleukins (IL) would be associated with a decreased risk of complicated appendicitis. On the contrary, we found that high concentrations of the Th2-associated IL-13 seemed to be associated with an increased risk of complicated appendicitis^13^. Other biomarkers, including a number of Th1/17 associated ILs, have been studied in the context of pediatric appendicitis, but their diagnostic accuracy in distinguishing uncomplicated from complicated cases remains unclear^14,15^. Additionally, certain biomarkers have only been investigated in adult cases of appendicitis and have not yet been studied in children^16,17^. In addition to elucidating its pathogenesis, a biomarker for complicated appendicitis in children could aid in clinical diagnostics which, in turn, would lead to better resource utility and health outcomes. Children with uncomplicated appendicitis could receive non-operative management^18^, thereby avoiding short- and long-term risks associated with surgery such as surgical site infections and small bowel obstructions^19,20^. In contrast, children with complicated appendicitis would be prioritized for timely surgical intervention. Ultrasound or computed tomography (CT) are frequently used when diagnosing appendicitis in children; however, both imaging techniques have limited ability to differentiate between uncomplicated and complicated appendicitis, particularly non-perforated complicated appendicitis^21–23^. Furthermore, CT exposes the child to ionizing radiation, increasing the risk of cancer^24,25^. Hence, the discovery of a biomarker for complicated appendicitis in children could have significant implications for the diagnosis, management and treatment of appendicitis. Thus, further research is needed to evaluate the utility of Th1/Th17 biomarkers in distinguishing uncomplicated from complicated appendicitis in pediatric patients. The aim of the present study was to evaluate how the Th1 and Th17-associated cytokines IL-1α, IL-1β, IL-2, IL-6, IL-10, IL-17A and tumor necrosis factor beta (TNF-β) are associated with the risk of complicated appendicitis in children.

## Methods

### Study design

A prospective cohort study was performed from 9 December 2017 to 16 February 2021. Patients were included at the Pediatric Emergency Department at Skåne University Hospital in Lund—a tertiary hospital with an uptake area of 350,000 inhabitants for general surgery emergencies. The study was approved by the Swedish Ethical Review Authority (Etikprövningsmyndigheten, Lund, Sweden, DNR 2013/614). The study was performed in accordance with relevant guidelines and regulations. Written parental informed consent was obtained from all included study subjects.

### Inclusion and exclusion criteria

All children aged < 15 years who were referred to a pediatric surgeon as a result of suspected appendicitis were eligible for inclusion. Children with a previous episode of suspected appendicitis, those with severe chronic diseases, and ongoing usage of anti-inflammatory drugs were excluded from the study.

### Data collection

Data were collected by the pediatric surgeon on call at the Pediatric Emergency Department. The following data were obtained: medical history, symptom duration, and findings on clinical examination. Study blood samples were collected only if clinical blood sampling was clinically indicated, in order not to cause unnecessary discomfort to the study participants. C-reactive protein (CRP), leukocytes, and neutrophiles were analyzed at the Department of Clinical Chemistry. Serum was saved and kept frozen until analyzes of cytokines was performed. Information on the final diagnoses, allergy status, results from the histopathological examination, and presence or absence of an appendicolith were obtained retrospectively from the medical records. Patients with appendicitis were diagnosed based on findings during surgery and histopathological examination, whereas patients diagnosed with other diagnoses were assessed clinically at the pediatric emergency department. The risk of missed appendicitis cases among the latter groups is considered low since the medical records were reviewed at least a couple of weeks after inclusion, and the same electronical medical record system is used at all emergency care facilities throughout the region.

### Analysis of cytokines

The serum separating tubes containing blood were left standing for 30 min before centrifugation at 2000G. Serum was then allocated to one to three test tubes, depending on the amount of serum available, containing 0.5 mL and frozen to − 80 °C. The frozen serum samples were stored at the regional biobank until analyzed with the Mesoscale Discovery^®^ (MSD, Maryland, USA) U-PLEX^®^ multiplex assay biomarker group (K15067L-2, MSD) with electro-chemiluminescence detection. To perform the analyzes, the U-PLEX™ multiplex SECTOR^®^ plate was prepared with biotinylated capture antibodies, 50 μL/well, for IL-1α, IL-1β, IL-2, IL-6, IL-10, IL-17A and TNF-β, and incubated overnight at 4 °C on a shaker. Calibrators 50 μL/well and serum (diluted 1:2) 50 μL/well were added after the plates had been washed three times with MSD wash buffer. A 1-h incubation at room temperature was followed by a new washing procedure and a SULFO-TAG™ detection antibody, 50 μL/well, was added. After a second 1-h incubation and a washing procedure, 150 μL MSD GOLD™ read buffer was added to each well was added and the plates were read on an MSD instrument. The intensity of emitted light was proportional to the concentration of cytokines present in the wells.

### Primary outcome, independent variables, and definitions

Primary outcome was complicated appendicitis. Primary exposures were serum concentrations of IL-1α, IL-1β, IL-2, IL-6, IL-10, IL-17A and TNF-β. Independent variables were age, sex, IgE-mediated allergy, symptom duration, and presence of an appendicolith. Symptom duration was estimated from reported onset of symptoms to the emergency visit. Diagnosis was determined by intraoperative findings and histopathological examination of extirpated appendices. Uncomplicated appendicitis was defined as phlegmonous appendicitis, histopathologically defined as infiltration of neutrophil granulocytes in the muscularis propria^2^. Complicated appendicitis was defined as gangrenous or perforated appendicitis, or the presence of an appendicular abscess^26^. Gangrenous appendicitis was defined as an inflammation of the appendix with necrotic discoloration as well as considerable tissue necrosis on histopathological examination without clinical signs of perforation^2^. Perforated appendicitis was defined as an observed hole in a gangrenous appendicitis or the finding of an appendicolith, free pus or intestinal content in the abdominal cavity^27^. Appendicular abscesses were diagnosed intraoperatively or radiologically. Appendicoliths were identified intraoperatively or preoperatively by means of ultrasonography or computed tomography.

### Statistics

IBM SPSS Statistics version 28.0 was used for all data analyzes. After data collection, children with missing data on all the biomarker concentrations were excluded from further analyzes. Continuous normally distributed data were presented as a mean with a 95% confidence interval (CI), continuous non-normally data were presented as median with interquartile range (IQR), and categorical variables were reported as frequencies and percentages. For comparison between groups in continuous normally distributed data the independent samples t-test was used. For comparison between two groups in continuous non-normally data, the Mann–Whitney U-test was used, and between three groups the Kruskal–Wallis test with a post hoc Dunn-Bonferroni test was used. For comparison between groups in categorical variables, the Chi-Squared test or Fisher’s exact test were used. Associations between Th1-related cytokines and the risk of complicated appendicitis were assessed using univariate and multivariable logistic regression, presented as odds ratio (OR) with 95% CI. Variables that were significantly associated with the risk of complicated appendicitis in the univariate logistic regression were included in the multivariable logistic regression.

## Results

**Figure 1:**
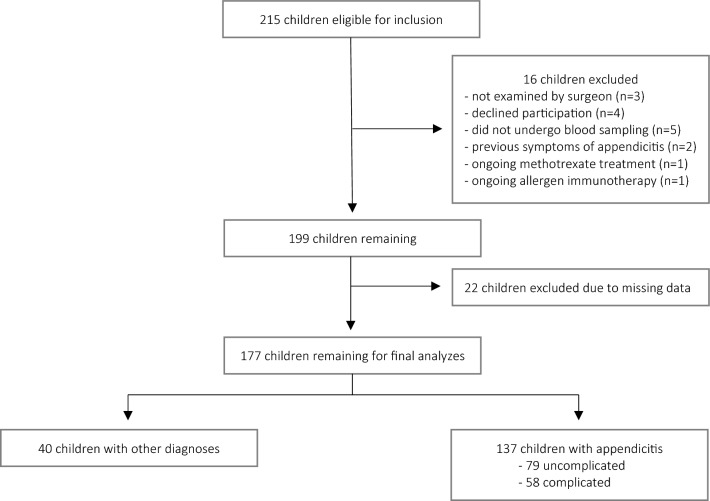
Flow chart of the inclusion and exclusion of 215 children with suspected appendicitis.

A total of 215 children were eligible for inclusion in the study. Sixteen children were excluded due to exclusion criteria. After excluding another 22 children as a result of missing data, a total of 177 children remained for analysis (Fig. 1). Of these, 137 (77%) children had appendicitis, of whom 79 (58%) had uncomplicated appendicitis and 58 (42%) had complicated appendicitis. The median age among children with appendicitis was 10 (IQR 8–12) years and 86 (63%) were boys (Supplementary Table 1). Children with complicated appendicitis were significantly younger (9 [IQR 7–12] years) and had a higher temperature (38.2 °C [95% CI 37.9–38.4]) compared to children with uncomplicated appendicitis (11 [IQR 9–13] years, *p* < 0.01 and 37.3 °C [95% CI 37.2–37.5], *p* < 0.01, respectively) (Table 1).

**Table 1:** Demographics of 137 children with appendicitis.

	Uncomplicated appendicitis n = 79	Complicated appendicitis n = 58	*p*-value
Age, years	11 (9–13)	9 (7–12)	** < 0.01**
Boys	48 (61%)	38 (66%)	0.57
Allergy	15 (19%)	5 (9%)	0.09
Temperature °C, mean	37.3 (37.2–37.5)	38.2 (37.9–38.4)	** < 0.01**
Symptom duration			**0.02**
< 24 h	41 (52%)	18 (31%)	
24–48 h	27 (34%)	23 (40%)	
48–96 h	10 (13%)	14 (24%)	
> 96 h	0 (0%)	2 (3%)	
CRP (mg/L)	26 (15–51)	57 (32–121)	** < 0.01**
Leukocytes (× 10^9^/L)	14.7 (13.5–15.8)	16.3 (15.1–17.5)	0.05
Neutrophiles (× 10^9^/L)	11.8 (10.7–12.9)	13.3 (12.2–14.4)	0.05

Continuous normally distributed data presented as mean (95% CI), continuous non-normally distributed data presented as median (IQR) and categorical variables presented as absolute n (%). Group differences were assessed through independent samples t-test for continuous normally distributed data, through Mann–Whitney U test for continuous non-normally distributed data and by Chi-square test for categorical data. Group differences were assessed through Fisher’s Exact Test for symptom duration.

Significant values are in bold.

**Figure 2:**
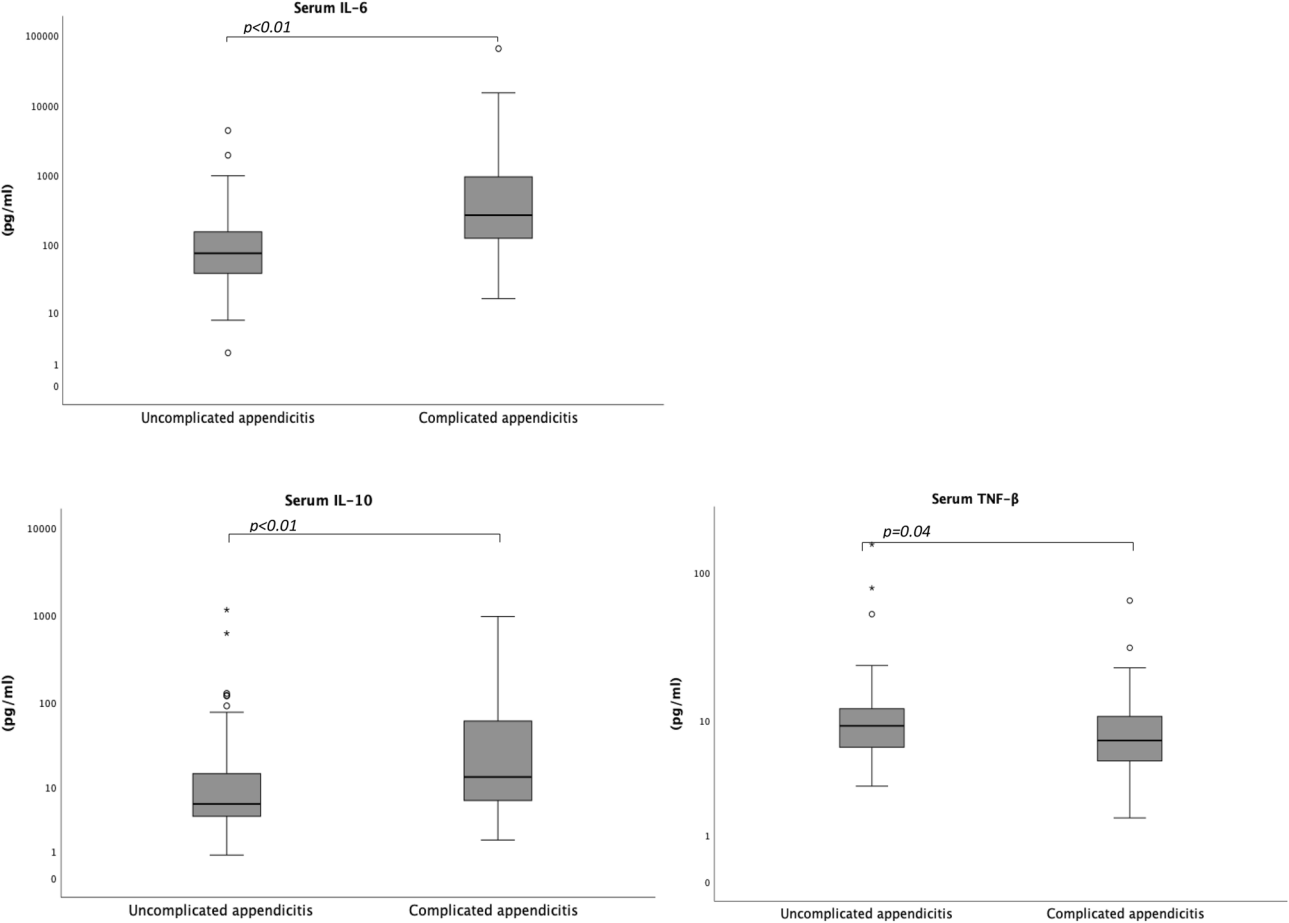
Concentrations of serum IL-6, IL-10 and TNF-β in 137 children with appendicitis.

The median concentration of serum IL-6 and IL-10 was significantly higher in children with complicated appendicitis (269.4 [IQR 122.4–953.8] pg/mL and 13.3 [IQR 6.4–69.3] pg/mL) compared to children with uncomplicated appendicitis (76.1 [IQR 38.6–167.3] pg/mL, *p* < 0.01 and 6.0 [IQR 4.1–14.8] pg/mL, *p* < 0.01, respectively). The median concentration of serum TNF-β was significantly lower in children with complicated appendicitis (7.2 [IQR 5.0–10.9] pg/mL) compared to children with uncomplicated appendicitis (9.2 [IQR 6.3–12.3] pg/mL, *p* = 0.04) (Table 2 and Fig. 2). The concentration of serum IL-1α, IL-1β, IL-2 and IL-17A did not differ significantly between children with uncomplicated and complicated appendicitis (Table 2).

**Table 2:** Serum concentrations of Th1/Th17-associated cytokines in 137 children with appendicitis.

	Uncomplicated appendicitis n = 79	Complicated appendicitis n = 58	*p*-value
IL-1α	164.7 (56.6–424.6)	37.8 (9.3–151.2)	0.18
IL-1β	5.3 (1.7–13.0)	6.6 (3.5–14.9)	0.20
IL-2	11.5 (1.4–48.4)	3.7 (0.3–12.4)	0.24
IL-6	76.1 (38.6–167.3)	269.4 (122.4–953.8)	** < 0.01**
IL-10	6.0 (4.1–14.8)	13.3 (6.4–69.3)	** < 0.01**
IL-17A	18.4 (11.7–34.2)	22.3 (15.0–42.5)	0.14
TNF-β	9.2 (6.3–12.3)	7.2 (5.0–10.9)	**0.04**

Values presented as median (IQR) (pg/mL), group differences assessed through Mann–Whitney U test. IL-1α n = 17 and 6. IL-1β n = 71 and 54. IL-2 n = 9 and 10. IL-10 n = 79 and 58. IL-17A n = 78 and 56. TNF-β n = 79 and 56.

Significant values are in bold.

**Table 3:** Unadjusted independent variables for complicated appendicitis in 137 children with appendicitis.

	Uncomplicated appendicitis n = 79	Complicated appendicitis n = 58	OR (95% CI)	*p*-value
Age, years	11 (9–13)	9 (7–12)	0.838 (0.746–0.943)	** < 0.01**
Boys	48 (61%)	38 (66%)	1.227 (0.606–2.484)	0.57
Allergy	15 (19%)	5 (9%)	0.403 (0.137–1.180)	0.10
Symptom duration				
< 24 h	41 (52%)	18 (31%)	Ref	Ref
24–48 h	27 (34%)	23 (40%)	1.940 (0.885–4.254)	0.10
48–96 h	10 (13%)	14 (24%)	3.189 (1.194–8.519)	**0.02**
> 96 h	0 (0%)	2 (3%)	N/A	N/A
Appendicolith	13 (17%)	21 (36%)	2.815 (1.256–6.309)	**0.01**
CRP (mg/L)	26 (15–51)	57 (32–121)	1.015 (1.007–1.024)	** < 0.01**
IL-1α (pg/mL)	164.7 (56.6–424.6)	37.8 (9.3–151.2)	0.994 (0.986–1.003)	0.19
IL-1β (pg/mL)	5.3 (1.7–13.0)	6.6 (3.5–14.9)	1.000 (1.000–1.001)	0.47
IL-2 (pg/mL)	11.5 (1.4–48.4)	3.7 (0.3–12.4)	0.970 (0.912–1.031)	0.32
IL-6 (pg/mL)	76.1 (38.6–167.3)	269.4 (122.4–953.8)	1.001 (1.000–1.002*)	**0.02**
IL-10 (pg/mL)	6.0 (4.1–14.8)	13.3 (6.4–69.3)	1.002 (0.999–1.005)	0.13
IL-17A (pg/mL)	18.4 (11.7–34.2)	22.3 (15.0–42.5)	1.009 (0.996–1.022)	0.18
TNF-β (pg/mL)	9.2 (6.3–12.3)	7.2 (5.0–10.9)	0.983 (0.951–1.016)	0.31

Values presented as median (IQR), and as n (%). Univariate logistic regression presented as odds ratios (ORs) with 95% confidence intervals (95% CI). N/A: not applicable. Symptom duration n = 78 and 57. Appendicolith n = 74 and 56. CRP n = 63 and 54. IL-1α n = 17 and 6. IL-1β n = 71 and 54. IL-2 n = 9 and 10. IL-10 n = 79 and 58. IL-17A n = 78 and 56. TNF-β n = 79 and 56. *1.000176–1.001810.

Significant values are in bold.

In the univariate logistic regression analysis, age (OR 0.84 [95% CI 0.75–0.94], *p* < 0.01), symptom duration (OR 3.19 [95% CI 1.19–8.52], *p* = 0.02), presence of an appendicolith (OR 2.82 [95% CI 1.26–6.31], *p* = 0.01), and concentration of serum IL-6 (OR 1.001 [95% CI 1.000–1.002], *p* = 0.02) were associated with an increased risk of complicated appendicitis. Serum concentrations of IL-1α, IL-1β, IL-2, IL-10, IL-17A and TNF-β were not associated with an increased risk of complicated appendicitis in the univariate logistic regression analysis (Table 3). The association between higher concentration of IL-6 and complicated appendicitis remained in the multivariable analysis after adjustment for age, symptom duration and presence of an appendicolith (OR 1.001 [95% CI 1.000–1.002], *p* = 0.02). Serum concentration of C-reactive protein (CRP) was also found to be associated with complicated appendicitis, even after adjustment for risk factors (OR 1.012 [95% CI 1.002–1.022], *p* = 0.02) (Table 4). The area under the receiver operating characteristic (ROC) curve (AUC) for IL-6 was 0.75 (95% CI 0.66–0.84) and for CRP was 0.73 (95% CI 0.64–0.82) (Supplementary Fig. 1).

**Table 4:**
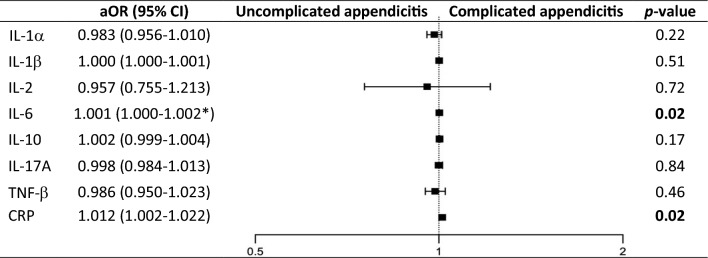
Adjusted variables for complicated appendicitis in 137 children with appendicitis

Multivariable logistic regression presented as adjusted odds ratios (aORs) with 95%confidence intervals (95%CI). Adjusted for age, symptom duration and presence of an appendicolith for each cytokine separately. IL-1α n=17 and 6. IL-1β n=71 and 54. IL-2 n=9 and 10. IL-10 n=79 and 58. IL-17A n=78 and 56. TNF-β n=79 and 56. CRP n=63 and 54.*1.000129–1.001660.

Among children without appendicitis, the most prevalent diagnosis was non-specified abdominal pain (Supplementary Table 1). Significant differences in serum concentrations of IL-6, IL-10, and TNF-β were observed when analyzing cytokine levels between the no appendicitis group, and the uncomplicated and complicated appendicitis groups (Supplementary Table 2 and Supplementary Fig. 2). In the univariate logistic regression analysis, male gender (OR 2.529 [95% CI 1.230–5.203], *p* = 0.01) and IL-17A (OR 0.990 [95% CI 0.980–1.000], *p* = 0.04) were associated with an increased risk of appendicitis (Supplementary Table 3).

## Discussion

This study aimed to evaluate the association between serum concentrations of Th1- and Th17-related cytokines with the risk of complicated appendicitis in pediatric patients. In our cohort, children with complicated appendicitis had significantly higher median concentrations of serum IL-6 and IL-10, and lower median concentrations of serum TNF-β compared to children with uncomplicated appendicitis. After adjusting for known risk factors such as age, symptom duration and the presence of an appendicolith in the multivariable regression analysis, higher concentrations of IL-6 remained associated with an increased risk of complicated appendicitis. For every 10-unit increase in IL-6, the adjusted risk of complicated appendicitis increased by 1%. Concentrations of IL-1α, IL-1β, IL-2, IL-10, IL-17A and TNF-β were not associated with an increased risk of complicated appendicitis in the adjusted regression analysis.

The association between higher concentrations of IL-6 and complicated appendicitis is in line with the results from previous studies^28–30^. IL-6 is a pro-inflammatory cytokine produced by a variety of cells including macrophages, T cells, endothelial cells and fibroblasts, and stimulates the synthesis of acute phase reactants such as CRP, fibrinogen and serum amyloid A^31^. Additionally, it is involved in the activation of B-cells and involved in the hematopoiesis. Although it is involved in the formation of Th17, it is not considered solely a Th17-associated cytokine as it is involved in multiple ways in the inflammatory response. IL-6 has been found to be elevated in several pathological circumstances, such as severe bacterial infections, sepsis, and in a variety of autoimmune diseases^32–34^.

Previous studies have found significantly higher levels of IL-10 in children and adults with complicated appendicitis^15,35^, which was confirmed in this study. However, there was no significant association between higher levels of IL-10 and complicated appendicitis in the logistic regression analysis. IL-10 was initially believed to be exclusively associated with Th2 cells; however, it has been shown to be produced by various cell types including Th1-cells. IL-10 is recognized as an anti-inflammatory cytokine as it operates through negative feedback on diverse immune responses^36^. The finding that IL-1β was not associated with an increased risk of complicated appendicitis is in accordance with a previous study on adults^16^, but in contrast to the results from a different study that found significantly higher levels of IL-1β in adults with complicated appendicitis^17^. Serum IL-17 has, in a previous study, been found to be elevated in adults with gangrenous appendicitis compared to adults with phlegmonous appendicitis^9^. However, IL-17 was not found to be associated with the risk of complicated pediatric appendicitis in the present study. The discrepancy in results may be explained by selection bias, small study sample sizes and different statistical methods. Additionally, it is important to remember that the immune system undergoes significant development during childhood, leading to potentially varying immune responses in children of different ages^37^. Hence, this may explain the discrepancy in results between different studies. It could be valuable to study the systemic inflammatory response in complicated appendicitis in a group of children with the same age, however, this would limit the generalizability of the findings to a specific age group.

In conclusion, it is possible that the systemic inflammatory response in uncomplicated versus complicated appendicitis is not as unambiguous as believed previously. Therefore, Th1/Th17-associated interleukins may not be appropriate prognostic tools for complicated appendicitis. Nevertheless, this study adds evidence supporting the previously indicated association between IL-6 and complicated appendicitis. However, the AUC for IL-6 was found to be 0.75, indicating only a moderate discriminatory ability in predicting complicated pediatric appendicitis, which is in line with previous studies that have found the AUC for IL-6 to be 0.70–0.77^28–30^. Studying the diagnostic property of IL-6 along with other diagnostic aids, such as clinical prediction scores, could offer valuable insights. Furthermore, future studies should focus on investigating the local inflammatory response within the appendix to enhance our understanding of the pathogenesis of uncomplicated and complicated pediatric appendicitis.

The results of this study need to be interpreted in the light of its limitations. One limitation in the present study is the rather small number of study subjects compared to other studies, and as a result we may not have sufficient power to exclude associations of certain cytokines. Additionally, since we did not have estimates of expected differences in the biomarker concentrations, no power analysis could be conducted. Although data were collected prospectively, 22 patients were excluded as a result of missing data, and this of course increases the risk of bias introduction. Another potential bias is that the concentrations of some of the biomarkers were unmeasurable and therefore missing for some patients. The main strength of the study is its prospective design, and that appendicitis diagnosis and severity were confirmed by histopathological examination. Furthermore, to our knowledge, some of the included biomarkers have not previously been evaluated regarding their association with complicated pediatric appendicitis.

## Conclusion

High serum concentrations of IL-6 were associated with an increased risk of complicated appendicitis in children, whereas serum concentrations of IL-1α, IL-1β, IL-2, IL-10, IL-17A and TNF-β were not. Hence, it is possible that the systemic inflammatory response in uncomplicated versus complicated appendicitis is not as unambiguous as believed previously. The AUC for IL-6 was found to be 0.75, indicating only a moderate discriminatory ability in predicting complicated pediatric appendicitis.

### Supplementary Information

Supplementary Information.

## Data Availability

Data supporting the results presented in this article will be made available by the corresponding author on reasonable request.
