# Supplementary material for: Associations between Th1-related cytokines and complicated pediatric appendicitis
Source: Sci Rep. 2024 Feb 26;14:4613. doi: 10.1038/s41598-024-53756-z (PMC10897334; doi:10.1038/s41598-024-53756-z)
Supplement: Supplementary file 1 — Supplementary Information. [file 41598_2024_53756_MOESM1_ESM.docx]

# Supplementary material


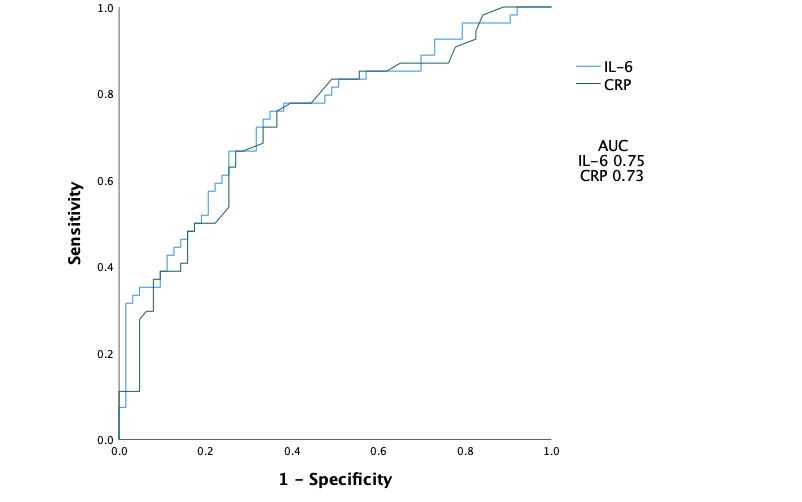


**Supplementary Figure 1.** Receiver operating characteristic (ROC) curve for IL-6 and CRP in predicting complicated appendicitis

**Supplementary Figure 2.** Concentrations of serum IL-6, IL-10 and TNF**-**β in a cohort of 177 children with suspected appendicitis


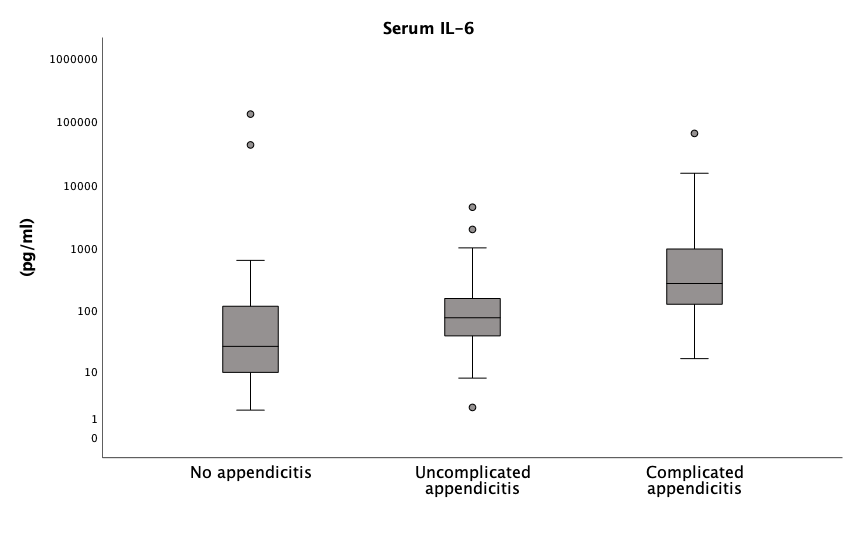


*p<0.01*

*p=0.03*

*p<0.01*


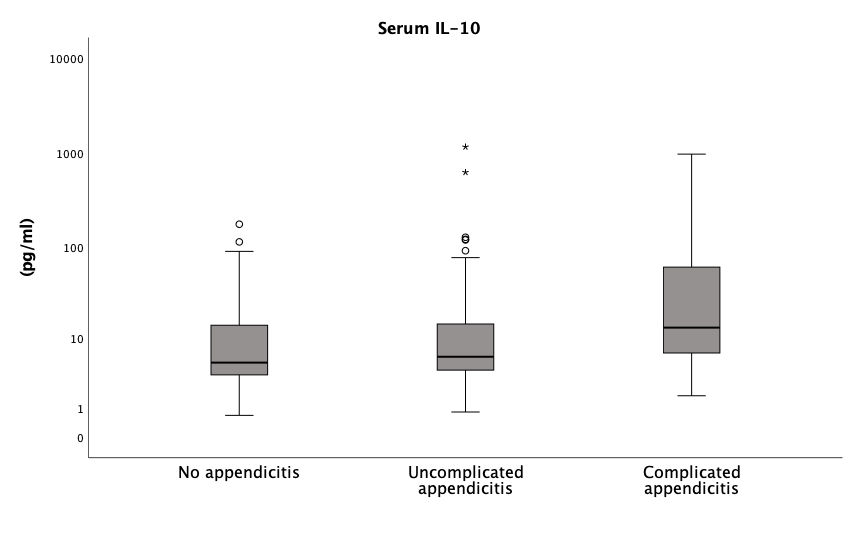


*p<0.01*

*p=0.43*

*p<0.01*


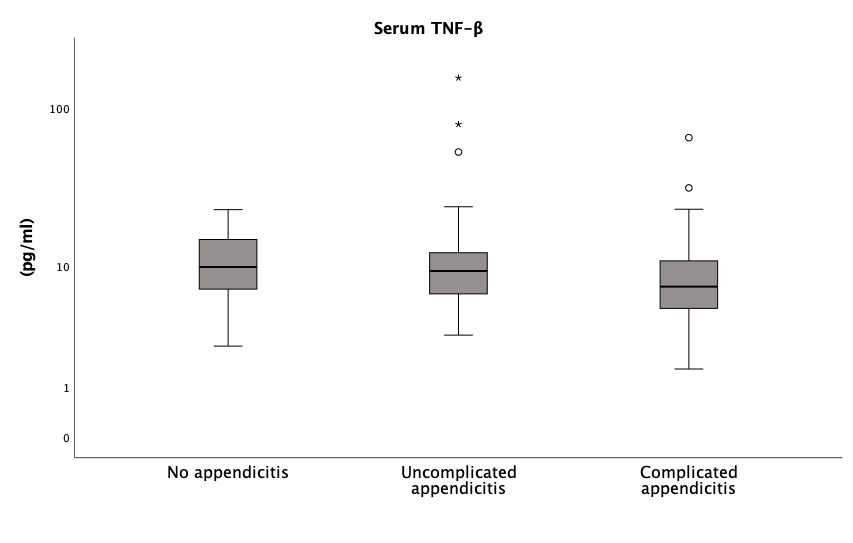


*p=0.02*

*p=0.46*

*p=0.05*

| **Supplementary Table 1.** Demographics and final diagnoses in 177 children with suspected appendicitis | | | |
| --- | --- | --- | --- |
|  | **No appendicitis**  **n = 40** | **Appendicitis**  **n = 137** | ***p*-value** |
| Age, years | 11 (9-13) | 10 (8-12) | 0.50 |
| Boys | 16 (40%) | 86 (63%) | **0.01** |
| Allergy | 3 (8%) | 20 (15%) | 0.24 |
| Temperature °C | 37.6 (37.3-38.0) | 37.7 (37.5-37.9) | 0.76 |
| Symptom duration 0-24 h  24-48 h  48-96 h  > 96 h | 14 (35%)  13 (33%)  9 (23%)  4 (10%) | 59 (43%)  50 (37%)  24 (18%)  2 (2%) | 0.08 |
| CRP (mg/L) | 34 (16-51) | 38 (20-85) | 0.31 |
| Leukocytes (x10^9^/L) | 11.3 (9.4-13.2) | 15.3 (14.5-16.2) | **<0.01** |
| Neutrophiles (x10^9^/L) | 8.3 (6.5-10.1) | 12.4 (11.7-13.2) | **<0.01** |
| Final diagnosis | Non-specified abdominal pain (26), Mesenterial lymphadenitis (3), Ovulation (2), Ileitis (1), Ovarial torsion (1), Pyelonephritis (1), Meckel’s diverticulum (1), Inguinal hernia (1), Constipation (1), Upper respiratory tract infection (1), Gastroenteritis (1), Abdominal tumor (1) | Phlegmonous (79)  Gangrenous (23)  Perforated (31)  Abscess (4) |  |
| *Data presented as mean (95% CI), median (IQR) or as n (%). Group differences were assessed through independent samples t-test for continuous normally distributed data, through Mann-Whitney U test for continuous non-normally distributed data and with Chi-square test for categorical data. CRP n=25 and 117. Leukocytes n=40 and 135. Neutrophiles n=39 and 134.* | | | |

| Supplementary Table 2. Serum concentrations of Th1/Th17-associated cytokines in 177 children with suspected appendicitis | | | | |
| --- | --- | --- | --- | --- |
|  | **No appendicitis**  **n = 40** | **Uncomplicated appendicitis**  **n = 79** | **Complicated appendicitis**  **n = 58** | ***p*-value** |
| IL-1α | 220.8 (90.0-828.1) | 164.7 (56.6-424.6) | 37.8 (9.3-151.2) | 0.08 |
| IL-1β | 17.6 (2.5-126.4) | 5.3 (1.7-13.0) | 6.6 (3.5-14.9) | 0.05 |
| IL-2 | 35.5 (2.9-47.7) | 11.5 (1.4-48.4) | 3.7 (0.3-12.4) | 0.21 |
| IL-6 | 26.2 (9.2-117.1) | 76.1 (38.6-167.3) | 269.4 (122.4-953.8) | **<0.01*** |
| IL-10 | 5.1 (3.5-15.1) | 6.0 (4.1-14.8) | 13.3 (6.4-69.3) | **<0.01**** |
| IL-17A | 22.8 (13.7-59.7) | 18.4 (11.7-34.2) | 22.3 (15.0-42.5) | 0.20 |
| TNF-β | 9.8 (6.6-14.9) | 9.2 (6.3-12.3) | 7.2 (5.0-10.9) | **0.04***** |
| *Values presented as median (IQR) (pg/mL), group differences assessed through Kruskal-Wallis test with a post hoc Dunn-Bonferroni test. Bold values indicate p-value below 0.05. IL-1α n=19, 17 and 6. IL-1β n=36, 71 and 54. IL-2 n=7, 9 and 10. IL-10 n=40, 79 and 58. IL-17A n=38, 78 and 56. TNF-β n=40, 79 and 56.  *Indicates significant differences between no appendicitis and uncomplicated appendicitis, between no appendicitis and complicated appendicitis, and between uncomplicated and complicated appendicitis.*  *** Indicates significant differences between no appendicitis and complicated appendicitis, and between uncomplicated and complicated appendicitis.*  **** Indicates significant difference between no appendicitis and complicated appendicitis.* | | | | |

| **Supplementary Table 3.** Unadjusted independent variables for appendicitis in 177 children with suspected appendicitis | | | | |
| --- | --- | --- | --- | --- |
|  | **No appendicitis**  **n=40** | **Appendicitis**  **n=137** | **OR (95% CI)** | ***p*-value** |
| Age, years | 11 (9-13) | 10 (8-12) | 0.965 (0.858-1.084) | 0.54 |
| Boys | 16 (40%) | 86 (63%) | 2.529 (1.230-5.203) | **0.01** |
| Allergy | 3 (8%) | 20 (15%) | 2.108 (0.593-7.496) | 0.25 |
| CRP (mg/L) | 34 (16-51) | 38 (20-85) | 1.004 (0.996-1.013) | 0.34 |
| IL-1α (pg/mL) | 220.8 (90.0-828.1) | 100.0 (20.0-305.3) | 0.999 (0.998-1.000) | 0.10 |
| IL-1β (pg/mL) | 17.6 (2.5-126.4) | 6.0 (0.6-13.8) | 1.000 (1.000-1.000) | 0.08 |
| IL-2 (pg/mL) | 35.5 (2.9-47.7) | 4.9 (0.6-16.5) | 0.995 (0.975-1.015) | 0.59 |
| IL-6 (pg/mL) | 26.2 (9.2-117.1) | 125.3 (59.2-336.8) | 1.000 (1.000-1.000) | 0.18 |
| IL-10 (pg/mL) | 5.1 (3.5-15.1) | 8.2 (4.5-27.5) | 1.006 (0.997-1.015) | 0.16 |
| IL-17A (pg/mL) | 22.8 (13.7-59.7) | 20.1 (12.6-36.1) | 0.990 (0.980-1.000) | **0.04** |
| TNF-β (pg/mL) | 9.8 (6.6-14.9) | 8.6 (5.5-11.9) | 1.005 (0.976-1.034) | 0.76 |
| *Values presented as median (IQR), and as n (%). Univariate logistic regression presented as odds ratios (ORs) with 95% confidence intervals (95% CI). CRP n=25 and 117. IL-1α n=19 and 23. IL-1β n=36 and 125. IL-2 n=7 and 19. IL-10 n=40 and 136. IL-17A n=38 and 134. TNF-β n=40 and 135.* | | | | |
